# Impact of Internet Addiction, Social Media Use and Online Pornography on the Male Sexual Function in Times of the COVID-19 Pandemic

**DOI:** 10.3390/jcm12196407

**Published:** 2023-10-08

**Authors:** Anna Pawlikowska-Gorzelańczyk, Daniel Fichte, Julia Rozmus, Piotr Roder, Remigiusz Flakus, Ewa Szuster, Kinga Brawańska, Małgorzata Biernikiewicz, Małgorzata Sobieszczańska, Agnieszka Rusiecka, Dariusz Kałka

**Affiliations:** 1 Cardiosexology Students Club, Wroclaw Medical University, 50-368 Wrocław, Poland; 2 Obstetrics and Gynecology Department, Wroclaw Medical University, 50-556 Wrocław, Poland; 3 Studio Słowa, 50-357 Wrocław, Poland; 4 Clinical Department of Geriatrics, Wroclaw Medical University, 50-369 Wroclaw, Poland; 5 Statistical Analysis Centre, Wroclaw Medical University, 50-367 Wroclaw, Poland; 6 Faculty of Physiotherapy, Wroclaw University of Health and Sport Sciences, 51-612 Wrocław, Poland; 7 Men’s Health Centre in Wrocław, 53-151 Wroclaw, Poland

**Keywords:** sexual dysfunction, addictive behavior, pornography, social isolation

## Abstract

Over recent decades, the use of the Internet has dramatically increased, both for professional purposes and entertainment. We investigated the link between social media use, video games, dating apps, and pornography on men’s sexual health, which makes life easier, but also carries potential threats. Online surveys including The International Index of Erectile Function (IIEF) and Bergen Social Media Addiction Scale questionnaires were spread to young, sexually active men. We asked about demographics, sexual activity, and the use of social media, video games, dating apps, and pornography. We enrolled 702 men aged 18 to 60 years (mean 24.06 ± 5.70). In general, 1.6% of men were exposed to social media addiction. Social media addiction had a negative impact on IIEF scores, while pornography in general had no impact on men’s sexual health. However, more extensive use of pornography was correlated with lower IIEF scores. A negative impact of dating apps use on the IIEF score was also found but the correlation was weak (*p* = 0.049). No correlation was found between playing games and IIEF. We conclude that social media addiction negatively affected men’s sexual functioning during the COVID-19 pandemic. The development of strategies for the safe use of the Internet and dissemination of this knowledge through social media campaigns can help young people to recognize the first symptoms of social media addiction.

## 1. Introduction

Over recent decades, the use of the Internet has dramatically increased. Today, about 5 billion people worldwide are Internet users [1]. In some European countries, around 99% of the population has an Internet connection. In Poland, it is around 87% [2]. As a consequence of the increased number of Internet users, the use of social media, video games, dating apps, and pornography has also increased. Nearly 60% of the global population are social media users [1]. There are 2.83 billion Facebook users and about 1.9 billion YouTube users. Platforms such as Instagram, Snapchat and TikTok are more popular among young people. Statistics show that daily hours spent with digital media increased from 2.7 h in 2008 to 6.3 h in 2018 [3]. Social media addiction concerns around 210 million people worldwide, while 34% of users claim that their absence on social media causes fear of missing out [4].

The same as social media, dating apps have become more accessible. Dating apps users are not only looking for a love or a sexual partner, but also want to meet new people. There are 276.9 million dating app users worldwide. The most popular portal is Badoo (105.73 million visits within one month), then Tinder (94.96 million visits) [5]. Furthermore, the access to the Internet is connected with the access to pornography. Around 40 million Americans regularly visit porn sites. Every second, 28,258 users are watching pornography on the Internet [6].

It is also remarkable that the number of gamers has increased. In 2022, 67% of Internet users played video games in comparison to 7% in 2014. Also over the years, gamers have undergone a steady transformation. Currently, gamers are a gender-balanced group. Many scientists have observe the negative impact of excessive use of the Internet on human health. In 1996, the definition of Internet addiction disorder was introduced [7]. Currently, an appellation “social networks use disorder” (SNUD) has been proposed [8]. Researchers have found a link between SNUD and the severity of depressive disorder, psychological distress and social anxiety [9]. Sexual health is strongly related to mental well-being [10]. Furthermore, the lockdown due to the COVID-19 pandemic caused an increased level of fear and loneliness [11]. The use of the Internet and social media has increased. Social media has become a facilitation but also a potential threat. On the one hand, social media gave an opportunity for online schooling, remote work, and having contact with family and friends. On the other hand, a high volume of mostly negative information in such a short period of time can cause mental overload [12]. 

The aim of our study was to investigate the impact of the social media use, online pornography and video games on men’s sexual health during the COVID-19 pandemic. The hypothesis posed in this work was the negative impact of excessive use of the Internet, social media, pornography and games on sexual functioning.

## 2. Materials and Methods

A self-reported questionnaire was spread to social media. The study group included 702 men aged 18 to 60 years. The questionnaire was based on the International Index of Erectile Function (IIEF), Bergen Social Media Addiction Scale (BSMAS) and additional sociodemographic questions. The answers were collected from May to July 2021. The sociodemographic interview included age, education, professional and marital status, presence of chronic diseases and their treatment. Respondents answered about their manner and time of using the Internet for professional and entertainment purposes, video games, and pornography. They were also asked about their attitude towards pornography and dating apps. 

The International Index of Erectile Function (IIEF) is a commonly used self-report questionnaire for the estimation of male sexual function and erectile dysfunction (ED) [13]. 

It includes 15 questions, concerns the last 4 weeks and examines 5 main domains: erectile function, orgasmic function, sexual desire, intercourse satisfaction, and overall satisfaction. Every answer is scored on a scale value of 0–5 (questions 1–10) and 1–5 (questions 11–15) [14]. Erectile function scores of IIEF total up to 30 points. ED was defined as a score of 25 or less.

BSMAS is a 6-item self-report scale that is a brief and effective psychometric instrument for assessing the risk for addiction to social media on the Internet [15]. The following aspects are examined: salience, mood modification, tolerance, withdrawal, conflict and relapse [16]. We used the Polish version of the scale [17]. Each question was rated on a 5-point Likert scale (1 = very rarely, 5 = very often), then the criterion was considered endorsed for the analyzes presented in this paper. In our study, the cutoff score was 24 points, which was suggested as optimal by Luo et al. [16].

Only questionnaires collected from sexually active men aged 18 years and older were analyzed. Participants were informed about the aim of the study, they could ask questions and the participation was voluntary. Each respondent gave his informed consent for participation in the survey. The questionnaire was verified with the CHERRIES checklist [18]. The study was approved by the Commission of Bioethics at Wroclaw Medical University, Wroclaw, Poland (KB-244/2023).

Statistics were analyzed methodically using Statistica software v. 13.3 (StatSoft, Tulsa, OK, USA). The answers were collected from the survey and illustrated by charts, numbers, percentages, standard deviations, medians, means and *p*-values, which perform the statistical significance of the test. The Shapiro–Wilk test was used, which determines whether the data system deviates from the Gaussian distribution. To compare all the variables between groups, the Mann–Whitney U test was used. The Kruskal–Wallis test with the post hoc median test was used when comparing more than two continuous variables. The differences were interpreted as statistically significant at *p* < 0.05. Cronbach’s alpha was used to assess the internal consistency of the questionnaire. A value higher than 0.7 indicates good internal consistency. This indicator was calculated for the entire questionnaire, covering all questions from the IIEF (15 questions) to the BSMAS (6 questions) sections. The results indicate that the questionnaire had good overall internal consistency, with a Cronbach’s alpha of 0.89. Internal consistency was also assessed separately for both parts of the questionnaire. The Cronbach’s alpha for the IIEF-15 and BSMAS parts was 0.94 and 0.84, respectively.

## 3. Results

In total, 702 men with a mean age of 24.06 years have been involved in our study. They were mostly students with secondary education, in relationships and inhabitants of big cities. The detailed data are presented in Table 1.

In the BSMAS questionnaire, 11 men (1.6%) received more than 24 points, which could be interpreted as a high risk of social media addiction. Detailed data are presented in Table 2. In the IIEF questionnaire, the mean score in the erectile function domain was 21.65 ± 9.88 (range 0–30), which can be interpreted as mild ED. What is more, the mean scores in orgasm (8.31 ± 3.37; range 0–10) and sexual desire (7.57 ± 1.65; range 0–10) were also related to mild dysfunction. However, intercourse satisfaction (8.25 ± 6.04; range 0–15) and overall satisfaction (6.49 ± 3.178; range 0–10) should be interpreted as mild to moderate dysfunction.

Analyzing the correlation between BSMAS and IIEF scores we concluded that there is a link between addiction to social media and ED (*p* = 0.02). The higher risk of social media addiction indicated more severe sexual dysfunction. Men who played video games were more likely to be at risk of Internet addiction (*p* = 0.003) than non-players. No correlation between IIEF and playing video games was found. The data are presented in Table 3.

**Figure 1:**
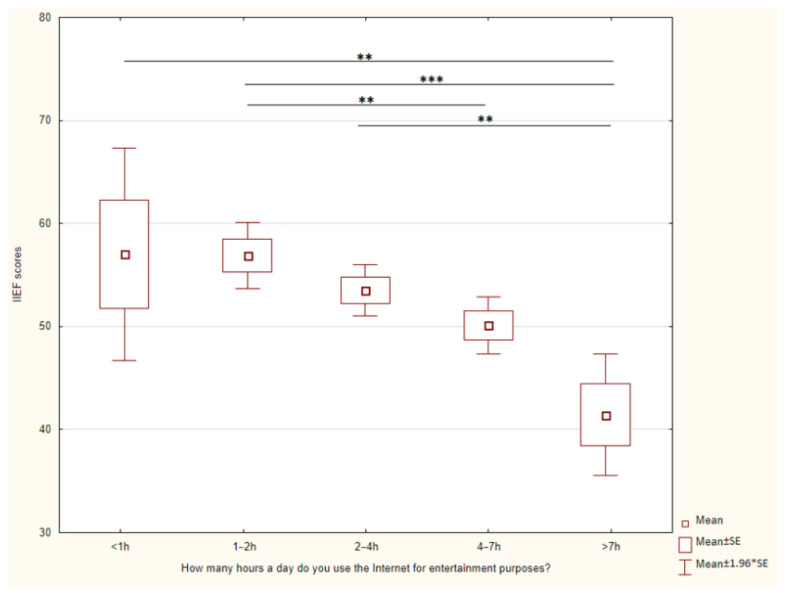
Relation between time spent on the Internet and IIEF scores. * *p* < 0.05, ** *p* < 0.01, *** *p* < 0.001.

A relation between time spent on the Internet and IIEF was found (Kruskal–Wallis, *p* < 0.001). There is a statistically significant difference between the groups of men depending on the time spent online. The detailed results are presented in Figure 1.

However, we found no effect of pornography use on the IIEF score (*p* = 0.75). Furthermore, we found that the IIEF score was significantly lower in dating apps users compared to the others (*p* < 0.05). The detailed data are presented in Table 4. 

More extensive pornography use was related with lower IIEF scores (Kruskal–Wallis, *p* = 0.04). The IIEF score was lower in the groups of people who used pornography everyday versus those who used pornography several times a year or less (*p* = 0.003) and versus those who used pornography a few times a week (*p* = 0.03). The largest groups of respondents answered that they used pornography a few times a week (38.03%), then several times a month (29.63%). We also found that a more positive attitude towards the internet pornography was related with better sexual functioning (Kruskal–Wallis, *p* < 0.001). Statistically significant differences were found between groups of single men and men in relationships. The detailed data are presented in Table 5.

We also analyzed the aims of Internet use and IIEF scores. YouTube, dating apps, and video games users had significantly lower IIEF scores. Science websites and on-line shopping were associated with better sexual functioning. We found no difference in social media use, remote work/online schooling or pornography use and sexual dysfunction. The detailed data are presented in Table 6.

**Figure 2:**
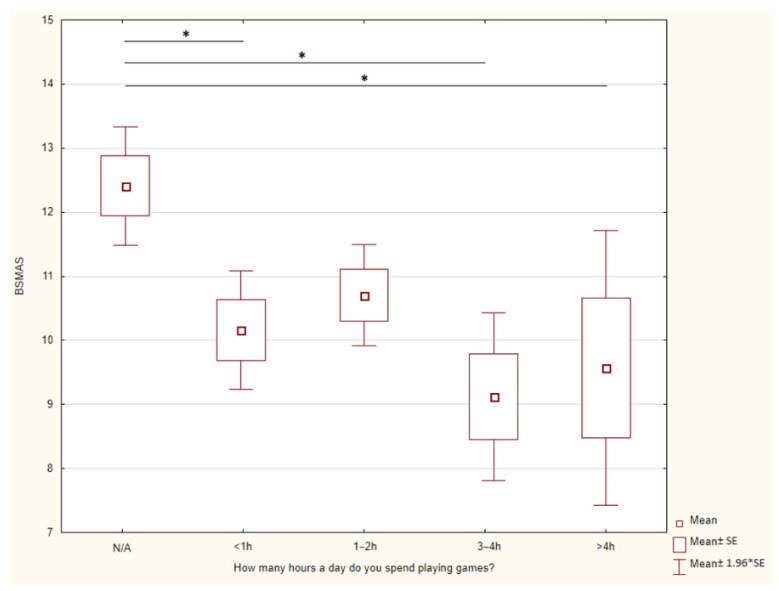
Time spent on playing games and BSMAS. * *p* < 0.05, ** *p* < 0.01, *** *p* < 0.001.

There are statistically significant differences depending on the time spent on playing games and BSMAS. Figure 2 presents this phenomenon.

We observed that only 177 (12.68%) of our respondents used dating apps, while the remaining 525 (74.79%) people denied using dating apps. The most popular aim of dating apps use was to find love (n = 37; 5.27%), then to meet new people (n = 29; 4.13%) and to find sexual partners (n = 23; 3.28%).

In summary, the results obtained confirm our hypothesis only partially. Social media addiction had a negative impact on IIEF scores, while pornography in general had no impact on men’s sexual health. A negative impact of dating apps use on the IIEF score was also found but the correlation was weak (*p* = 0.049). No correlation was found between playing games and IIEF.

## 4. Discussion

The feeling of loneliness and isolation during the COVID-19 pandemic caused excessive use of the Internet and social. It could intensify the consequences of excessive social media use and lead to addiction and consequently induce sexual dysfunction. 

Today, social media has become an integral part of daily life. People use various platforms to have permanent contact with the world, friends, and family members. Taking it a step further, social networks are becoming not only a way to communicate with friends, but also a place where everyone can shop, sell unnecessary items, and find discount codes. Moreover, portals such as Instagram or TikTok are the source of inspiration. Many users look for ideas what to cook, how to furniture the apartment, what to wear, and where to go on holidays. There is also an opportunity to look into the private lives of celebrities, as well as old friends or ex-partners. That makes it very common for social media users to compare themselves with others. On the other hand, social media gives the opportunity to create their own, imagined life by posing appropriate pictures, so it seems to be perfect. This is the reason why more and more people feel unhappy, have complexes, and have lowered self-esteem. About 90% of people aged 18–29 years claim to use social media in any available form, which means that they are at the highest risk of becoming addicted to social media [4]. The results of our study show that social media addiction is a common problem with 1.6% of our respondents being at high risk of social media addiction, 37.18% of men using the Internet more than 4 h daily, and 8.83% of men feeling that excessive social media use has a negative impact on their relationship. Rogowska et al. revealed that 17.19% of Polish students are addicted to Instagram [19]. It is curious to note that in Rogowska et al. research the adverse consequences of Instagram addiction on life satisfaction were identified specifically among female students, while no such effect was observed among their male counterparts. Our study indicates a correlation between social media addiction and decreased level of sexual health. Sexual health is influenced by many factors such as sexual behavior, attitudes, societal factors, mental, emotional and physical state [20]. Among respondents who are addicted to social media or Internet, a higher prevalence of depressive symptoms was revealed [21]. The literature review conducted by Holland et al. shows increased body dissatisfaction and increased prevalence of eating disorders correlated with time spent online. In their study, the authors highlighted particular behaviors, such as the act of viewing and sharing photos and actively seeking negative feedback through status updates, as being especially detrimental to body image and their potential impact on the development of eating disorders. [22]. Our research also showed the link between free time spent online and the IIEF score. The more time spent on the Internet, the lower level of sexual health was observed. On the other hand, time spent online for professional purposes had no impact on men’s sexual health. It can be assumed that the changes in daily life caused by excessive social media use could lead to ED. However, it should be considered that our study was conducted during the COVID-19 pandemic. Lockdown, prolonged self-isolation, remote work and online studies, which could also escalate the Internet addiction. Limited perspectives for the free-time activities may lead to more extensive use of social media and Internet. Furthermore, young adults occur to be at increased risk of COVID-19 fear [23]. A study conducted in Poland revealed that fear of the pandemic could lead to emotional distress and depressive disorders, particularly among young people [24]. Depression, as well as other consequences of the pandemic like andrological complications could also lead to ED [25]. 

Another part of the Internet use is watching pornography. Pornography has become a normative behavior among adults. The percentage of men watching pornography is definitely noticeable. Almost 90% of our respondents used online pornography and 10% watched it on a daily basis. However, no correlation was found between pornography use and IIEF. Berger et al. revealed that men use pornography more frequently than women (81.1% VS. 39%). Men who preferred partnered sex without pornography had a lower risk of ED in comparison to men who preferred masturbation to pornography over partnered sex [26]. However, Jacobs et al. investigated the correlation between pornography addiction and IIEF-5 and concluded that higher scores indicated problematic online pornography consumption leading to a higher probability of ED [27]. In our study, we also revealed that more extensive pornography use was correlated with lower IIEF scores (*p* = 0.04).

Currently, the initiation of interpersonal relationship begins with the “swiping to the right”. Dating Apps are becoming an integral part of many people these days. Apps such as Tinder report 1.4 billion swipes per day, helping 6 billion friendships, professional partnerships, and platonic relationship matches, and 26 million matches daily for people aged 18 to 50 years. However, many stereotypes and stigmas about dating apps are still being observed. Many people consider dating apps as a place where they can find a partner for one night stands [28]. In recent years, many studies have investigated why people use dating apps. It should be noted that the use of such applications is not only related to the sexual sphere. Up to 70% of users declare non-sexual purposes such as building a social network, finding a romantic partner, traveling (having dates in different places), self-validation and entertainment [29]. The results of our study show that 52.21% of men use dating apps, 41.6% of them are looking for love, 25.8% are looking for a sexual partner, and 32.6% of men are looking for new friends. In addition, men who use dating apps have lower IIEF scores and more often declare watching pornography. 

In recent years, the use of video games has increased, which is likely associated with advancements in technology and the improved quality of computer games being produced. In our study, 75.5% of the respondents were gamers. An equally high percentage of respondents indicated that they played both war and strategy games (43.3%), with arcade games following closely (31.2%). However, no link between BSMAS and IIEF was found. Sansone et al. also indicated that differences between gamers and non-gamers in the domains of erectile function, orgasmic function, and overall satisfaction were not significant [30]. 

The strength of our research is that it is the first study which examines this problem in Poland. Its novelty relies on examining the link between social media use, pornography and dating apps and sexual dysfunction. Our results may help sexologists and psychiatrists in the diagnostic process and therapy of the abovementioned dysfunction. The purpose of our study was also to show what kind of consequences an excessive use of the Internet and social media can involve, and to point out how important it is to properly educate the society on this topic. Our research shows that excessive use of the Internet and social media affects men’s sexual life. The COVID-19 pandemic exacerbated this situation. Social media addiction is a serious problem and therefore physicians should pay attention to this issue. A special program should be introduced to inform young people about the consequences and risks of social media and Internet addiction. The youth should be taught from an early age about the risks of excessive use of social media. Parents, who have an integral influence on the development of their children, should also be educated. Young people need to know strategies for coping with this problem. Furthermore, sexual education programs in schools should include information about pornography and dating apps.

Our study has some limitations that should be kept in mind while analyzing the results. First of all, our respondents were in general young men and, respectively, our study is not representative of the whole male population. What is more, both IIEF and BSMAS are self-reported questionnaires and our findings should not be interpreted as a professional psychiatric diagnosis. In addition, we have to highlight that depressive and sexual disorders could also be caused by stress related to the pandemic, chronic diseases, relationship with the partner, etc. Due to socio-demographic reasons, our research is limited to developed countries, such as Poland, where access to the Internet is facilitated. On the other hand, in underdeveloped countries, the lack or reduction of Internet access will result in a decreased time spent on social media, pornography, etc. Our questionnaire did not contain questions about unhealthy diet or sedentary lifestyle, which can also lead to men’s sexual disorders.

## 5. Conclusions

Addiction to social media has a negative impact on male sexual function. We found no link between pornography use and ED while dating apps had a negative impact on IIEF scores. The increased use of pornography was correlated with worse sexual functioning. Video games players showed a stronger predilection to Internet addiction in comparison to non-players. The more positive attitude towards Internet pornography was correlated with better sexual health. Development of strategies for the safe use of the Internet and dissemination of this knowledge through social media campaigns can help young people to recognize the first symptoms of social media addiction.

## Figures and Tables

**Table 1:** Characteristics of the study group.

Variable	Result; N = 702
Age, years; Mean ± SD	Mean 24.06 ± 5.70
Education	
Primary	16 (2.28%)
Vocational	11 (1.57%)
Secondary	412 (58.69%)
Higher	263 (37.46%)
Employment status	
Student	415 (58.53%)
Employed	259 (36.53%)
Unemployed	13 (1.83%)
Other	15 (2.12%)
Marital status	
Single	252 (35.90%)
Married	62 (8.83%)
In partnership	388 (55.27%)
Place of living	
Rural area	133 (18.95%)
City >50,000 inhabitants	92 (13.11%)
City from 50,000 to 100,000 inhabitants	51 (7.26%)
City from 100,000 to 250,000 inhabitants	72 (10.26%)
City above 250,000 inhabitants	354 (50.43%)
Comorbid chronic disease	
No	537 (76.50%)
Yes	165 (23.50%)
On treatment due to any disease	
No	224 (31.91%)
Yes	478 (68.09%)
How many hours do you spend online at work?	
<1 h	135 (19.23%)
1–2 h 2–4 h 4–7 h >7 h	131 (18.66%) 173 (24.64%) 155 (22.08%) 108 (15.38%)
How many hours do you spend online to relax? <1 h 1–2 h 2–4 h 4–7 h >7 h	20 (2.85%) 141 (20.09%) 280 (39.89%) 209 (29.77%) 52 (7.41%)

**Table 2:** Results of the Bergen Social Media Addiction Scale.

Question		Never (0 Points)	Very Rarely (1 Point)	Rarely (2 Points)	Sometimes (3 Points)	Often (4 Points)	Very Often (5 Points)
	Answer						
1. Thinking about social media		155 22.08%	135 19.23%	233 33.19%	117 16.67%	45 6.41%	17 2.42%
2. Feeling an urge to use social media more		149 21.23%	113 16.10%	160 22.79%	162 23.08%	77 10.97%	41 5.84%
3. The use of social media to forget about personal problems		135 19.23%	141 20.09%	184 26.21%	139 19.80%	82 11.68%	21 2.99%
4. No success in cutting down on social media use		275 39.17%	109 15.53%	162 23.08%	98 13.96%	44 6.27%	14 1.99%
5. Feeling restless if social media using is prohibited		226 32.19%	161 22.93%	125 17.81%	109 15.53%	55 7.83%	26 3.70%
6. Too extensive use of social media has a negative impact on job or studies		117 16.67%	98 13.96%	169 24.07%	192 27.35%	93 13.25%	33 4.70%

**Table 3:** Correlations between the questionnaires and playing computer games.

	Response	IIEF Mean ± SD	IIEF Median (Q1–Q3)	*p*-Value
BSMAS	0–24 pkt (n = 691)	52.49 ± 20.94	63 (32–69)	0.02
	25–29 pkt (n = 11)	38.00 ± 22.79	29 (17–63)	
Do you play computer games?	Yes (n = 582)	51.90 ± 21.19	63 (31–69)	0.13
	No (n = 120)	54.05 ± 21.18	64 (34–70)	
		BSMAS Mean ± SD	BSMAS Median (Q1–Q3)	
Do you play computer games?	Yes (n = 582)	10.53 ± 6.20	10 (6–15)	0.003
	No (n = 120)	12.40 ± 6.47	12.5 (7–17)	

**Table 4:** IIEF in relation to the use of dating apps and pornography.

Domain	Response	IIEF, Mean ± SD	*p*-Value	Chi-Square
Do you use dating apps?	Yes (n = 177)	42.04 ± 21.61	0.049	12.12
	No (n = 525)	55.71 ± 19.68		
Do you use pornography?	Yes (n = 630)	52.36 ± 20.82	0.75	
	No (n = 72)	51.47 ± 22.92		

**Table 5:** IIEF versus frequency of pornography use, attitude to the pornography and marital status.

	**Response**	**IIEF** **Mean ± SD**	**IIEF** **Median (Q1–Q3)**	***p*** **-Value**
Do you use pornography?	Yes, a few times a year or less (n = 83)	58.37 ± 18.67	67 (57–70)	0.04
	Yes, a few times a month (n = 208)	53.27 ± 20.34	64 (32–69)	
	No (n = 72)	51.47 ± 22.92	61.5 (30.5–71)	
	Yes, a few times a week (n = 267)	51.38 ± 20.75	63 (31–69)	
	Yes, everyday (n = 72)	46.40 ± 23.10	59.5 (26–68)	
What is your attitude to internet pornography?	1: very negative (n = 76)	44.43 ± 23.80	45 (19.5–67.5)	<0.001
	2: negative (n = 86)	48.47 ± 20.07	56.5 (31–67)	
	3: neutral (n = 258)	52.05 ± 21.47	64 (31–69)	
	4: positive (n = 184)	53.65 ± 19.93	64 (33–69)	
	5: very positive (n = 98)	59.64 ± 17.74	67 (59–71)	
Are you in a relationship?	No (n = 252)	33.64 ± 18.88	30 (18.5–38)	<0.001
	Yes, I’m married (n = 62)	63.03 ± 13.12	67 (63–70)	
	Yes, I’m in informal relationship (n = 388)	62.65 ± 13.79	67 (63–70)	

**Table 6:** IIEF versus aims of Internet use.

What are Your Main Aims of Internet Use?	Response	IIEF Mean ± SD	IIEF Median (Q1–Q3)	*p*-Value
YouTube or watching movies and TV series online	Yes (n = 648)	51.87 ± 21.03	63 (31.5–69)	0.03
	No (n = 54)	57.02 ± 20.67	67 (46–71)	
Social media (Facebook, Twitter, Instagram)	Yes (n = 572)	52.11 ± 21.14	63 (31.5–69)	0.69
	No (n = 130)	52.95 ± 20.61	63 (33–69)	
Video games	Yes (n = 344)	51.17 ± 21.13	62 (31–68)	0.04
	No (n = 358)	53.32 ± 20.91	64.5 (33–70)	
Dating apps	Yes (n = 65)	36.88 ± 21.05	31 (18–62)	<0.001
	No (n = 637)	53.84 ± 20.40	64 (34–69)	
Science websites and apps	Yes (n = 347)	54.28 ± 20.05	65 (34–69)	0.04
	No (n = 355)	50.30 ± 21.80	61 (30–69)	
Remote work/online schooling	Yes (n = 451)	51.28 ± 20.83	62 (31–69)	0.07
	No (n = 251)	54.03 ± 21.29	64 (33–70)	
Online shopping	Yes (n = 264)	54.91 ± 19.78	65 (35–70)	0.02
	No (n = 438)	50.68 ± 21.62	62 (30–69)	
Pornography	Yes (n = 369)	52.22 ± 20.73	63 (32–68)	0.53
	No (n = 333)	52.32 ± 21.39	63 (31–70)	

## Data Availability

The data that support the findings of this study are available from the authors on request.
